# Irreversibly increased nitrogen fixation in *Trichodesmium* experimentally adapted to elevated carbon dioxide

**DOI:** 10.1038/ncomms9155

**Published:** 2015-09-01

**Authors:** David A. Hutchins, Nathan G. Walworth, Eric A. Webb, Mak A. Saito, Dawn Moran, Matthew R. McIlvin, Jasmine Gale, Fei-Xue Fu

**Affiliations:** 1 Marine and Environmental Biology, Department of Biological Sciences, University of Southern California, 3616 Trousdale Parkway, Los Angeles, California 90089, USA; 2 Marine Chemistry and Geochemistry Department, Woods Hole Oceanographic Institution, 266 Woods Hole Road, Woods Hole, Massachusetts 02543, USA

## Abstract

Nitrogen fixation rates of the globally distributed, biogeochemically important marine cyanobacterium *Trichodesmium* increase under high carbon dioxide (CO_2_) levels in short-term studies due to physiological plasticity. However, its long-term adaptive responses to ongoing anthropogenic CO_2_ increases are unknown. Here we show that experimental evolution under extended selection at projected future elevated CO_2_ levels results in irreversible, large increases in nitrogen fixation and growth rates, even after being moved back to lower present day CO_2_ levels for hundreds of generations. This represents an unprecedented microbial evolutionary response, as reproductive fitness increases acquired in the selection environment are maintained after returning to the ancestral environment. Constitutive rate increases are accompanied by irreversible shifts in diel nitrogen fixation patterns, and increased activity of a potentially regulatory DNA methyltransferase enzyme. High CO_2_-selected cell lines also exhibit increased phosphorus-limited growth rates, suggesting a potential advantage for this keystone organism in a more nutrient-limited, acidified future ocean.

## Abstract (web summary)

The long-term response of marine cyanobacteria to increased anthropogenic CO_2_ are not known. Here, Hutchins *et al*. show that *Trichodesmium* exposed to long-term selection at elevated CO_2_ display irreversible increases in nitrogen fixation and growth rates, even after returning to present day conditions.

## Graphical abstract

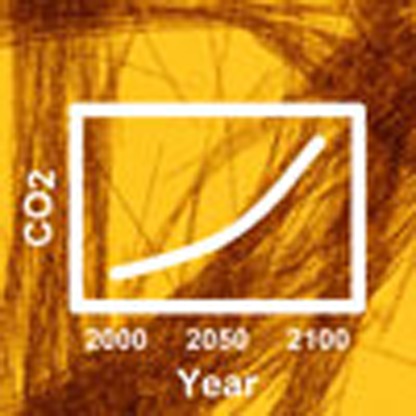

Atmospheric dinitrogen (N_2_) fixation by marine cyanobacteria is a globally dominant source of the limiting nutrient nitrogen to the ocean's biosphere^1^. The colony-forming genus *Trichodesmium* is among the most important contributors of newly fixed nitrogen to marine food webs, with some estimates suggesting it may carry out as much as half of total N_2_ fixation in the vast subtropical gyre biomes of the ocean^2^. Due to the pivotal role that N_2_-fixing cyanobacteria play in the ocean's nitrogen cycle, environmental factors such as phosphorus, iron and light that can potentially limit *Trichodesmium* N_2_ fixation rates have been the subject of intensive study^3^,^4^,^5^,^6^.

A number of recent studies have demonstrated that carbon dioxide (CO_2_) may also limit the N_2_ fixation rates of marine cyanobacteria, including *Trichodesmium*. Enhancement of *Trichodesmium* N_2_ fixation rates by up to 50% at projected year 2100 CO_2_ atmospheric levels (∼800 p.p.m.) relative to present day concentrations (∼400 p.p.m.) points to the possibility that new nitrogen inputs from this cyanobacterium could greatly increase as a result of future anthropogenic CO_2_ emissions^7^,^8^,^9^,^10^,^11^. Such a CO_2_ fertilization effect on marine N_2_ fixation would fundamentally change the nitrogen cycle of the ocean^12^. However, most of these previous studies were based on results from only one or two cultured isolates. A recent examination of a diverse global collection of N_2_-fixing cyanobacteria offers a more nuanced viewpoint, whereby particular strains, species and perhaps clades appear to be optimized for growth and N_2_ fixation under either low or high CO_2_ conditions. This observation of taxon-specific CO_2_ niche specialization suggests that marine N_2_-fixing cyanobacteria may have undergone differential selection by the many spatial and temporal CO_2_ fluctuations they have experienced during their long evolutionary history^8^.

Although this indirect evidence suggests that adaptation may be key to understanding the response of *Trichodesmium* to future changes in atmospheric CO_2_, none of these previous studies were designed to evaluate its potential evolutionary trajectories under high CO_2_. All previous work with *Trichodesmium* used only brief exposures to elevated CO_2_, typically on the order of weeks^7^,^9^,^10^. This is long enough to determine transient acclimation physiology, but inadequate to ascertain the types of long-term evolutionary responses *Trichodesmium* may exhibit during the up to 19,000 generations that it will have to adapt to rising CO_2_ levels over the next century.

To address this issue, here we employ classic experimental evolution methods that provide insights into the fundamental principles of microbial adaptive responses^13^,^14^,^15^,^16^,^17^,^18^. We grow six replicate cell lines of *Trichodesmium erythraeum* strain IMS 101 for 4.5 years at 380 p.p.m. (current CO_2_, ∼570 generations) or 750 p.p.m. (projected year 2100 CO_2_, ∼850 generations), and then evaluate changes in their reproductive fitness using growth rate measurements, as well as changes in their physiological rates of N_2_ fixation. Every replicate cell line selected under expected future high-CO_2_ conditions exhibits elevated N_2_ fixation and growth rates that are constitutively expressed, in that they remain fixed at high levels despite reciprocal transfers back to long-term growth under lower current CO_2_ concentrations. This surprising microbial evolutionary response is characterized by apparently permanent changes in diel periodicity of N_2_ fixation and increased expression of a DNA-methylating enzyme with a possible regulatory function, and persists even when growth rates are limited by the nutrient phosphorus. *Trichodesmium* reacts to projected anthropogenic CO_2_ increases not only through transitory physiological plasticity responses, but also in wholly unexpected adaptive ways that could have large consequences for nitrogen and carbon biogeochemical cycling in the future ocean.

## Results

### Experimental evolution at 380 and 750 p.p.m. CO_2_

**Figure 1:**
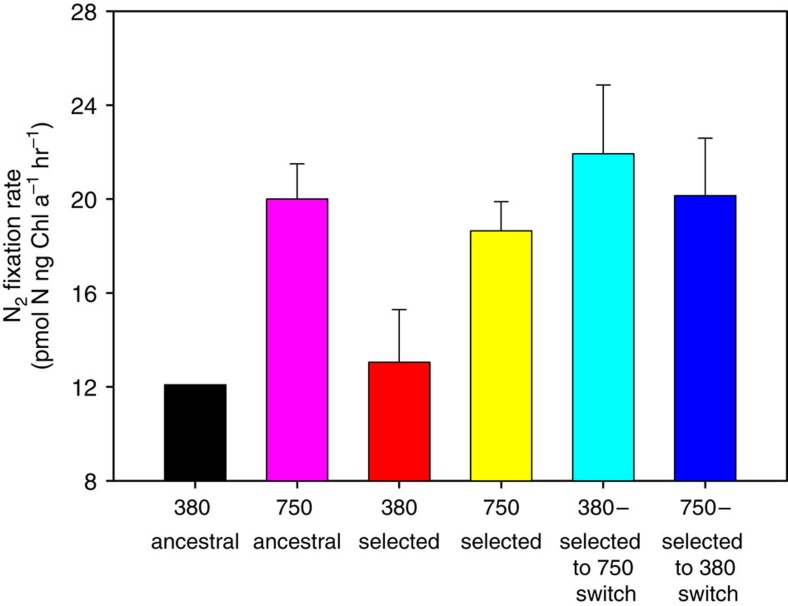
*Trichodesmium* N_2_ fixation rates before and after long-term selection at 380 or 750 p.p.m. CO_2_. The ancestral cell line grown at 380 p.p.m. CO_2_ (black) and 750 p.p.m. CO_2_ (pink) before the selection experiment; and at the end of the 4.5 year CO_2_ selection period in 380-selected (red) and 750-selected (yellow) cell lines, as well as in these same selected cell lines 2 weeks after reciprocal transfers (380-to-750-switch, turquoise; and 750-to-380-switch, blue). Values are the means and error bars are the s.d. of six replicate cell lines for each treatment.

Before the experimental evolution study, the ancestral cell lines exhibited a large increase in N_2_ fixation rates when stock cultures maintained at 380 p.p.m. CO_2_ (380 ancestral) were transferred to 750 p.p.m. CO_2_ (750 ancestral) for 2 weeks (Fig. 1, *P*<0.05, Student's *t*-test, *n*=6), as expected based on the previous short-term experiments cited above. At the end of the 4.5 year selection period, N_2_ fixation rates of the six 380- and 750-selected cell lines were not significantly different from those of the ancestral lineage at these same respective CO_2_ levels before selection (Fig. 1, *P*>0.05, Student's *t*-test, *n*=6). N_2_ fixation rates of the 750-selected cell lines were still 43% higher than those of the 380-selected cultures (Fig. 1a, *P*<0.05, Student's *t*-test, *n*=6). This is consistent with the responses of the ancestral cell line (Fig. 1), and published short-term studies^7^,^9^,^10^.

We then tested for any adaptive changes arising due to selection under the two CO_2_ regimes. Following the 4.5 year selection period, we conducted short-term reciprocal transfers to the opposite CO_2_ level for both the 380- and 750-selected cell lines^16^,^17^. When subcultures of the six 380-selected cell lines were transferred to 750 p.p.m. CO_2_ for 2 weeks (380-selected to 750 switch, Fig. 1), they rapidly increased their N_2_ fixation rates to levels very similar to those in the 750-selected cultures (Fig. 1).

### High CO_2_ irreversibly increases N_2_ fixation and growth

**Figure 2:**
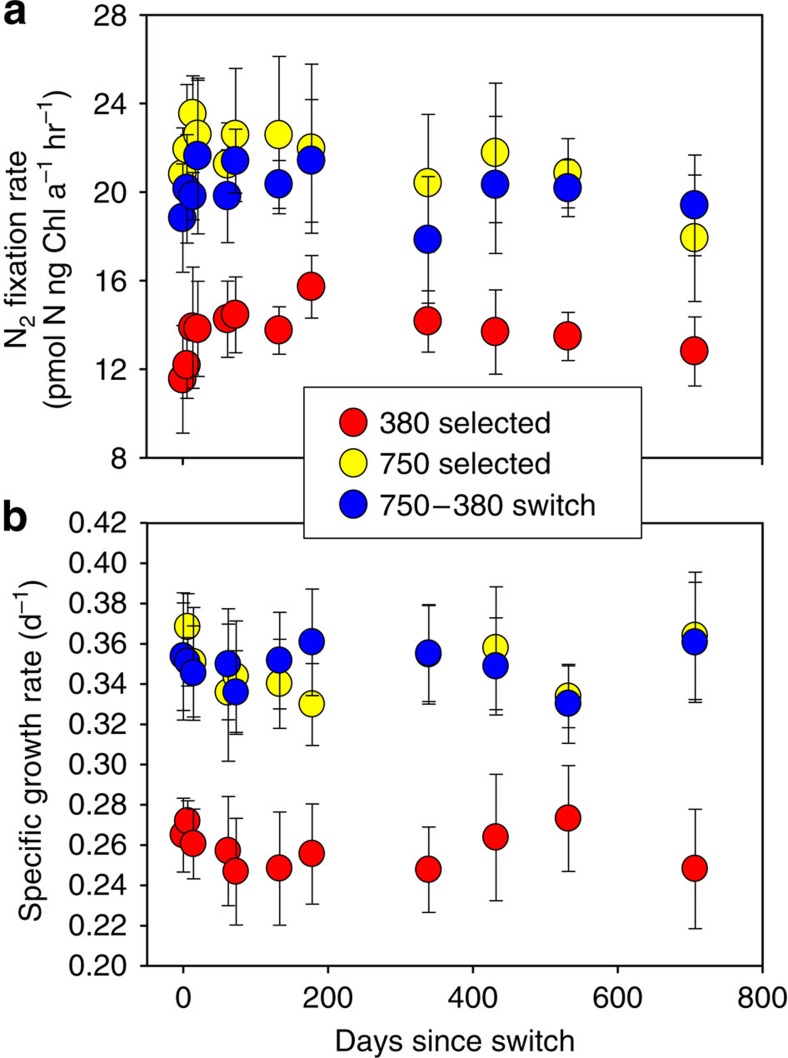
Irreversibly increased *Trichodesmium* N_2_ fixation and growth rates following long-term selection by high CO_2_. (**a**) N_2_ fixation rates in the 750-to-380 switch cell lines (blue) over the subsequent ∼2 years following the reciprocal transfer, while being maintained at the switched CO_2_ condition of 380 p.p.m. Also shown are N_2_ fixation rates of the long-term 750-selected (yellow) and 380-selected (red) cell lines maintained over the same time period continuously at 750 and 380 p.p.m., respectively (**b**) Cell-specific growth rates of the same cell lines and treatments shown in panel (**a**). Data points represent the means, and error bars the s.d. of six replicate cell lines for each treatment.

Unexpectedly though, the 750-selected cell lines returned to 380 p.p.m. CO_2_ for 2 weeks did not show a corresponding decrease in their N_2_ fixation rates (750-selected to 380 switch, Fig. 1). Instead, their N_2_ fixation rates persisted at very high levels, indistinguishable from those of the 750-selected and 380-selected to 750 switch cultures (*P*>0.05). These same six 750-selected to 380 switch cell lines were then maintained at the reciprocal CO_2_ level of 380 p.p.m. over the subsequent ∼2 years, along with the long-term 750-selected and 380-selected cell lines maintained at their respective selection CO_2_ levels. N_2_ fixation rates of the 750-selected cultures switched back to 380 p.p.m. for 2 years (>350 generations) remained virtually identical to those of the 750-selected cell lines maintained continuously at 750 p.p.m., with both still ∼43% higher relative to rates of the 380-adapted cell lines maintained at 380 p.p.m. (Fig. 2a, *P*<0.05, Student's *t*-test, *n*=6). Thus, every cell line selected at 750 p.p.m. CO_2_ was unable to reduce their rates when moved back to the ancestral 380 p.p.m. CO_2_ level, with N_2_ fixation physiology instead becoming ‘stuck in the fast lane'.

Cell-specific growth rates of all six replicate cell lines responded in the same way as N_2_ fixation rates. 750-selected to 380 switch cultures maintained at 380 p.p.m. for 2 years grew at the same rates as the 750-selected cell lines maintained continuously at 750 p.p.m.; both grew up to ∼44% faster than the 380-selected cultures maintained at 380 p.p.m. (Fig. 2b, *P*<0.05, Student's *t*-test, *n*=6). Thus as with N_2_ fixation rates, the growth rates of all six 750-selected cell lines became constitutively elevated relative to CO_2_ concentration following 4.5 years of selection at high CO_2_, with the same universally increased growth rates persisting regardless of whether they were grown at 380 or 750 p.p.m.

### Irreversibly shifted diel N_2_ fixation patterns

**Figure 3:**
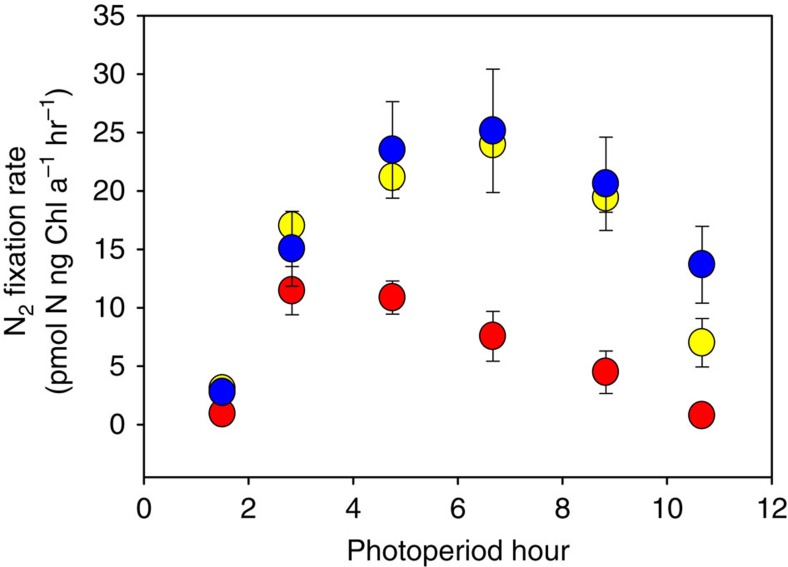
Diel periodicity of N_2_ fixation in 380-selected, 750-selected and 750-selected-to-380-switch *Trichodesmium* cell lines. Shown are N_2_ fixation rates across the 12-h photoperiod in cell lines selected for ∼6.5 years at 380 p.p.m. (red) or 750 p.p.m. (yellow), and in the cell lines selected at 750 p.p.m. for ∼4.5 years and then transferred back to 380 for ∼2 years (blue). Data points represent the means, and error bars the s.d. of six replicate cell lines for each treatment.

An examination of diel patterns of N_2_ fixation in the selected cell lines offers possible insights into the physiological mechanisms behind this unexpected adaptive response to prolonged growth under high CO_2_. *Trichodesmium* fixes nitrogen only during daylight hours, with rates often peaking somewhere near mid-photoperiod^19^. Our 380-selected cell lines conformed to this expected pattern, with the highest N_2_ fixation rates occurring between 3 and 5 h into the photoperiod (Fig. 3). In both the 750-selected and 750-selected to 380 switch cell lines, however, N_2_ fixation rates were not only higher, but the peak fixation period was nearly doubled in duration and shifted to later in the photoperiod (hours 5–9, Fig. 3). A similar shift in diel N_2_ fixation patterns has been reported in experiments using *Trichodesmium* grown for shorter periods under high CO_2_ (ref. 20), but our results demonstrate that this altered periodicity is an integral feature of the observed irreversible effects of long-term adaptation to elevated CO_2_. Despite being moved back to 380 p.p.m. for ∼2 years, the 750-selected to 380 switch cell lines maintained a diel N_2_ fixation pattern nearly identical to that of the 750-selected cell lines, with higher absolute fixation levels occurring over a longer period of time later in the photoperiod (Fig. 3).

### Proteomics and DNA methyltransferase activity

**Figure 4:**
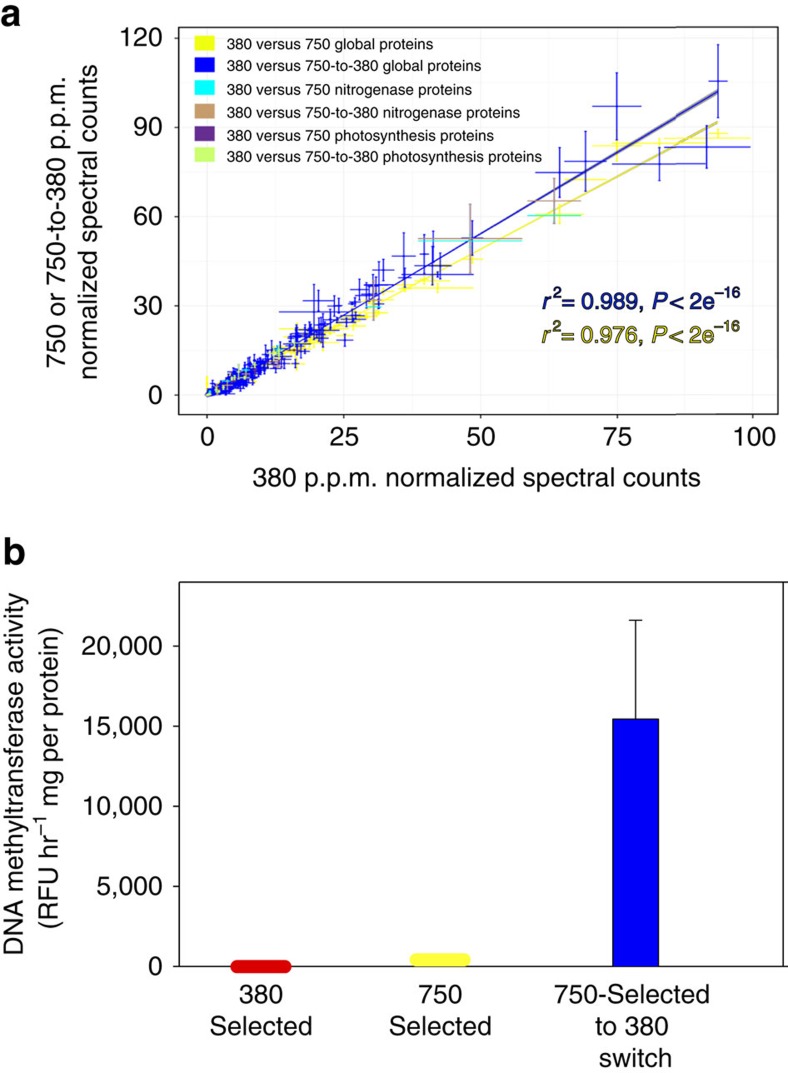
Relative expression levels of ∼1,500 proteins, and DNA methyltransferase enzyme activities in the 750-selected to 380 switch, 750-selected and 380-selected cell lines. (**a**). Global protein expression levels with linear regressions are plotted for the 380-selected cell lines versus the 750-selected (yellow symbols) and versus the 750-selected to 380 switch (blue symbols) cell lines. Regressions indicate that both relationships are not significantly different from 1:1. Highlighted in colour are expression levels of proteins associated with the N_2_-fixing nitrogenase enzyme complex (380-selected versus 750-selected, turquoise symbols; 380-selected versus 750-selected to 380 switch, brown symbols) and with cellular photosynthetic proteins (380-selected versus 750-selected, purple symbols; 380-selected versus 750-selected to 380 switch, light green symbols); these also fall on the regression lines and are not significantly different from 1:1. Symbols are the means and error bars are the standard errors of three replicate cell lines for each treatment (**b**). Relative DNA methyltransferase enzyme activity (relative fluorescence units per hour per mg cellular protein) 20 months after the reciprocal transfer switch. Shown are enzyme activity levels in the 380-selected (red), 750-selected (yellow) and 750-selected to 380 switch (blue) cell lines. All data points represent the means, and error bars the standard errors of six replicate cell lines for each treatment.

Proteomic analyses of samples taken near the mid-point of the photoperiod from three of the biological replicates from each treatment did not show evidence for differential expression of the ∼1,500 distinct proteins detected in the 750-selected and 750-selected to 380 switch cell lines, relative to the 380-selected cell lines (Fig. 4a). In particular, proteins typically associated with elevated N_2_ fixation and growth such as those comprising the nitrogenase enzyme complex and photosynthetic systems were detected, but were not more highly expressed in the two high CO_2_-selected cell lines (Fig. 4a). This lack of a definitive change in expression of proteins at mid-photoperiod, even those intimately involved in N_2_ fixation, suggests that the observed constitutive rate increases following selection at high CO_2_ may be due to subtle changes in regulatory controls on N_2_ fixation activity, rather than to more obvious quantitative alterations in the proteome.

Accordingly, regulatory mechanisms such as DNA methylation and protein phosphorylation have been shown to control numerous cellular processes and genetic networks in bacteria, including the cell cycle, gene expression, motility, DNA repair, heritable phenotypic variation and adaptation to novel environments^21^,^22^,^23^. To investigate potential regulatory changes in our CO_2_-selected *Trichodesmium* cell lines, we tested the activity of DNA methyltransferase enzymes ∼20 months following the switch by fluorometrically measuring the total amount of methyl groups transferred to cytosine on a DNA substrate (Methods section). Methyltransferase activity levels were elevated to varying degrees in all six 750-selected to 380 switch cell lines, but were below detection or nearly so in every one of the 380-selected and 750-selected cell lines (Fig. 4b). These data suggest that transferring 750-selected cell lines back to 380 could result in increased levels of methyltransferase-mediated regulation, which could potentially be related to the observed constitutive rate increases and associated irreversible shifts in diel N_2_ fixation patterns. This regulatory enzymatic response, like elevated growth and N_2_ fixation, has now persisted for more than 350 generations following the switch back to low CO_2_.

### High CO_2_ adaptation and phosphorus limitation

**Figure 5:**
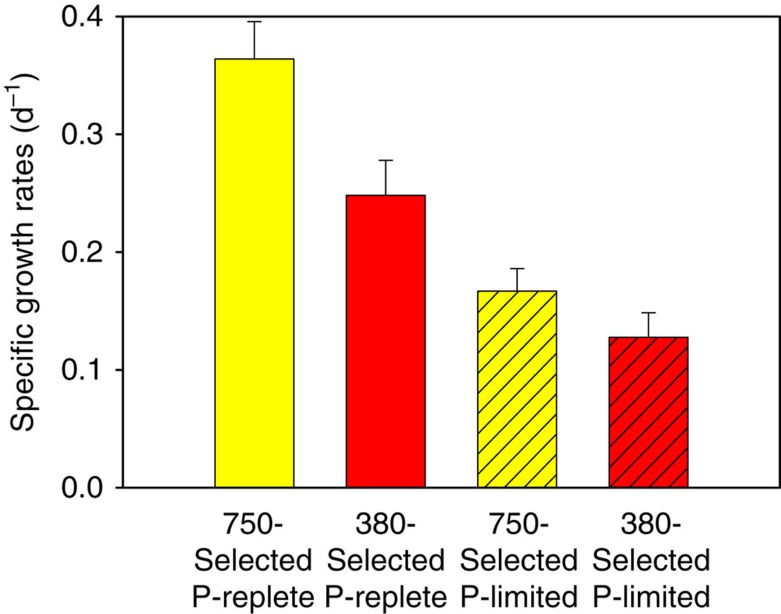
Phosphorus-limited growth rates of CO_2_-selected *Trichodesmium* cell lines. Cell-specific growth rates (d^−1^) of the 750- and 380-selected cell lines grown in nutrient-replete medium (yellow and red bars, respectively) and for the 750- and 380-selected cell lines grown in phosphorus (P)-limited medium (hatched yellow and red bars, respectively). Values are the means, and error bars are the s.d. of six replicate cell lines for each treatment.

Phosphorus is one of the key limiting nutrients for N_2_ fixation in the ocean^1^,^3^,^5^. To examine interactions between phosphorus availability and adaptation to increasing CO_2_, we cultured our six 750-selected and 380-selected cell lines for 2 months under phosphorus-limited and phosphorus-replete conditions at their respective CO_2_ selection levels (Methods section). Phosphorus-limited growth rates of both sets of cell lines were lower than phosphorus-replete rates, as expected (Fig. 5a, *P*<0.0001, Student's *t*-test, *n*=6). In phosphorus-replete cultures, the 750-selected cell lines grown at 750 p.p.m. had growth rates that were much higher than those of the 380-selected cell lines growing at 380 p.p.m. (Fig. 5a, *P*<0.0001, Student's *t*-test, *n*=6), again as expected from previous results (Fig. 1a). Notably though, the 750-selected cell lines were still able to grow ∼1.3 times faster than the 380-selected cell lines when both were grown under identical phosphorus-limited conditions (Fig. 5, *P*<0.007, Student's *t*-test, *n*=6).

## Discussion

*Trichodesmium* growth rates, a proxy for microbial reproductive fitness^13^,^14^, increased almost immediately when cell lines were moved to 750 p.p.m. CO_2_, but then remained unchanged despite the subsequent ∼850 generations of selection at this elevated CO_2_ level. Thus, beyond the previously documented rapid initial physiological plasticity response, long-term selection by high CO_2_ did not lead to further measurable fitness increases in the selection environment. However, these same 750-selected cell lines exhibited a 44% fitness increase when transplanted back to the ancestral CO_2_ condition of 380 p.p.m., relative to the 380-selected and ancestral cell lines. This surprising adaptive response–in which short-term plastic growth rate increases become fixed during extended selection in a novel environment, resulting in permanent large fitness increases even in the ancestral environment–appears to be unique in the microbial experimental evolution literature^13^,^14^.

Changes in evolutionary fitness often show tradeoffs in relative fitness across different environments^13^,^14^,^24^. An evolution experiment using a freshwater green alga showed no fitness increases in a high CO_2_ selection environment, and some but not all high CO_2_-selected cell lines exhibited fitness decreases when switched back to ambient CO_2_ concentrations^18^. Analogous to our *Trichodesmium* cell lines, in another study measuring *Escherichia coli* long-term evolutionary adaptation to several temperature regimes all cell lines improved fitness relative to their ancestors in the selection environment. However, these fitness increases occurred only following thousands of generations of exposure, and replicate *E. coli* cell lines demonstrated considerable heterogeneity in whether fitness was decreased or unchanged at ancestral temperatures; none exhibited fitness increases in the ancestral environment^25^. Our *Trichodesmium* results differ markedly from these prior results, in that within a few hundred generations all six biological replicates of our high CO_2_-selected cell lines evolved a homogeneous, correlated phenotype. This phenotype consisted of permanent retention of fitness increases originally acquired due to a physiological plasticity response, and that conferred increased fitness even in the ancestral environment.

Regardless of the ultimate mechanisms, our findings could have major ecological and evolutionary implications for these biogeochemically critical cyanobacteria in the future high-CO_2_ ocean. Rapidly growing, high CO_2_-selected *Trichodesmium* might appear to possess a potential competitive advantage over other primary producers; however, if faster growth rates are universally advantageous, it is remarkable that our 380-selected cell lines did not also acquire the same adaptation that each of our 750-selected cell lines did. Indeed, it is questionable why wild *Trichodesmium* populations growing at current CO_2_ levels have not adapted to grow at similarly high rates. It is evident that there must be tradeoffs involved with maintaining constitutively elevated growth and N_2_ fixation rates that make them intrinsically maladaptive at present day CO_2_ concentrations.

Although the nature of these tradeoffs is currently unknown, one potential disadvantage with elevated growth rates is that they result in increased cellular demand for limiting resources such as nutrients that are in short supply over large parts of the ocean^3^,^4^,^5^,^6^,^26^,^27^. Indeed, pervasive nutrient limitation is likely to be exacerbated in the future oligotrophic oceans. Surface warming and freshening are predicted to intensify density-driven stratification, further reducing vertically-advected sources of phosphorus and other nutrients^28^,^29^. Our results suggest that high CO_2_-selected *Trichodesmium* may enjoy a competitive advantage in the future more stratified and nutrient-limited ocean, and that this advantage is also likely to persist, even if they are subsequently returned to lower CO_2_ conditions. However, since limitation by iron and simultaneous co-limitation by both iron and phosphorus are also ecologically important^1^,^2^,^3^ and elicit distinctive physiological and growth responses in *Trichodesmium*^6^,^30^, it will be important to determine interactions of these nutrient conditions with long-term selection by high CO_2_ as well.

Our cell lines were grown in seawater medium with CO_2_ levels controlled artificially by bubbling, which maintained partial pressures within ±<5% of our target values of 380 and 750 p.p.m. (Methods section). In the oligotrophic ocean where *Trichodesmium* grows, CO_2_ concentrations in contemporary surface waters vary over a similar range around current atmospheric levels of ∼400 p.p.m. (refs 31, 32). Presumably, CO_2_ concentrations will range within similar bounds around projected year 2100 atmospheric levels of ∼750 p.p.m., suggesting that our results are likely applicable to seawater carbonate chemistry conditions that will be experienced by *Trichodesmium* in future surface waters of the subtropical central gyre ecosystems. It is also necessary, however, to examine to what extent laboratory culture results such as ours can be extrapolated to a comprehensively changing future ocean environment, where many factors other than CO_2_ will be simultaneously in flux.

Some of the physiological responses to long-term selection by high CO_2_ we documented in our *Trichodesmium* experimental evolution cell lines have been previously observed following brief acclimation periods in short-term experiments, including elevated N_2_ fixation and growth rates, even under phosphorus-limited conditions, and shifts in diel N_2_ fixation patterns^7^,^9^,^10^,^20^. Our remarkable finding is that these same phenotypes became irreversibly fixed in all six cell lines that experienced extended selection under projected future CO_2_ conditions. The fact that this unprecedented evolutionary response to selection by elevated CO_2_ occurs in a microbe that plays such a critical role in global biogeochemistry suggests potentially far-reaching consequences for the ocean's nitrogen cycle under accelerating anthropogenic change. It also raises another long-term environmental question. How and when will future high CO_2_-selected *Trichodesmium* strains be able to re-adapt to lower surface ocean CO_2_ levels, assuming our species is eventually able to curb our global consumption of fossil fuels?

It is evident that even a relatively well-studied organism like *Trichodesmium* can still present us with evolutionary surprises when faced with a single global change factor like higher CO_2_. Future changing environmental conditions in the ocean's vast central gyre biomes will include not only higher CO_2_, but also warmer temperatures, lower nutrient inputs, higher irradiance exposures, expanding hypoxia, and novel competitive and trophic interactions within altered biological communities^33^,^34^. Predicting the net adaptive responses of keystone marine functional groups like N_2_-fixing cyanobacteria to the integrated effects of this entire complex matrix of changing environmental variables remains a daunting challenge for the future.

## Methods

### Culturing *Trichodesmium* cell lines

Cultures of *Trichodesmium* strain IMS 101 obtained from the National Center for Marine Algae and Microbiota (NCMAA, Bigelow Laboratory for Marine Sciences, East Boothbay Harbor, Maine 04544, USA) were maintained in a modified Aquil medium^35^,^36^ with standard mixed vitamins and trace metals, containing 500 nM iron and 20 μM phosphate but without combined nitrogen under a light intensity of 120 μmol photons per metre square per second with a light–dark cycle of 12:12 high:dark in 26 °C incubators. Six replicate cell lines were used for each treatment to provide robust statistical confidence in the experimental evolution results^14^,^15^. Semi-continuous dilution culturing methods were practiced in this experiment because they allow measurement of CO_2_ effects during acclimated, steady-state growth. The cultures were kept optically thin to avoid self-shading, nutrient limitation and perturbations of targeted CO_2_ levels. Each bottle was diluted individually based on the growth rate calculated for that bottle^7^,^8^,^11^,^36^. Growth rates were calculated according to microscopic cell counts for reported values, or using *in vivo* chlorophyll fluorescence measurements with a Turner 10 AU fluorometer for semi-daily dilution calculations in real time during the experiments. Comparisons between cell counts and *in vivo*-based growth rates revealed no significant differences between the two methods of assessing biomass changes.

Experimental evolution cultures were continuously bubbled with prepared air/CO_2_ mixtures (Praxair) to maintain stable CO_2_ concentrations of 380 p.p.m. or 750 p.p.m. CO_2_ for ∼4.5 years. At this time, a set of short-term 2 week CO_2_ reciprocal transfer incubation experiments was performed using the long-term cultures. These two treatments consisted of switching 380 p.p.m. CO_2_-conditioned cell lines to 750 p.p.m. CO_2_, and 750 p.p.m. CO_2_-conditioned cultures to 380 p.p.m. CO_2_ (referred to as switch cultures). The switch incubations were performed under experimental conditions and dilution frequencies identical to those of the long-term cultures.

Following the 2 week reciprocal transfers, the six 750 to 380 switch cell lines were then maintained for a further 2 years at 380 p.p.m., in parallel with the 750-selected and 380-selected cell lines maintained at their relative selection CO_2_ levels. During this subsequent 2 year period, all culturing protocols for the switch and long-term selected cultures remained the same as outlined above. The classical method to determine fitness changes in microbial experimental evolution experiments is competition of selected cell lines against the ancestral strain^13^,^14^,^15^. This is not possible for *Trichodesmium*, though, since it is not amenable to cryopreservation. Consequently, we used the best available measurable indicator of relative fitness, specific growth rates, to assess adaptive changes, with the caveat that this proxy may not fully capture all fitness changes in our CO_2_-selected *Trichodesmium* cell lines. For all experiments, significant differences between the six replicates in each treatment were tested using one-way Anova followed by student's *t*-test.

### Phosphorus limitation experiments

The six replicate long-term 750-selected and 380-selected *Trichodesmium* cell lines were used in experiments assessing the responses of cell-specific growth rates to phosphorous limitation. Medium was prepared for P-replete cultures as described above for experimental cultures, and was prepared in the same way except the phosphate concentration was reduced to 0.5 μM for P-limited cultures. Steady-state growth rates of the semi-continuous cultures were measured after 2 months of growth at 26 °C in the P-limited medium, and results are reported as the means and averages of the six replicate cell lines for each treatment. P limitation cultures were maintained in nutrient-limited exponential growth by transferring them every 2 days using semi-continuous culturing methods, so cultures were never allowed to deplete medium nutrients or reach stationary phase^30^. Our P limitation results thus represent cells in rate-limited ‘Blackman' limitation, rather than biomass-limited ‘Liebig' limitation^37^.

### N_2_ fixation measurements

Nitrogen fixation rates were measured using the acetylene reduction assay with a Shimadzu gas chromatograph GC-8a (Shimadzu Scientific Instruments, Columbia, Maryland) equipped with a flame ionization detector. A theoretical ratio of 3:1 (mol C_2_H_2_:mol N_2_ reduced) was used to convert rates of ethylene production (C_2_H_2_ reduction) to N_2_ fixation. Assays were initiated by adding 2 ml of C_2_H_2_ to the headspace of 28-ml serum vials containing 10 ml of culture. A measure of 100 μl headspace was removed to measure ethylene production at 2–3 h intervals over the entire 12-h light period. Samples were gently agitated to equilibrate gas concentrations between the headspace and culture samples following injection of acetylene and before measuring ethylene concentrations^7^,^8^,^11^,^36^.

### Carbonate buffer system measurements

To ensure that target selection CO_2_ levels were correct, the seawater carbonate buffer system was analysed in the experimental bottles periodically throughout the entire experiment, including at each sampling point. pH was measured using a Orion 5 STAR pH meter (Thermo Fisher Scientific) with a combined glass electrode. The metre was calibrated with National Bureau of Standards buffer solutions of pH 4, 7 and 10. Dissolved inorganic carbon was measured with CO_2_ coulometry (model CM 140, UIC). The partial pressure of CO_2_ in the samples was calculated from the measured pH and dissolved inorganic carbon values using CO_2_SYS software^7^,^8^,^11^. Because pCO_2_ equilibrium was closely controlled using continuous bubbling, photosynthesis and respiration had minimal effects on the seawater carbonate system and measured pCO_2_ was always within ∼5% of the two target values.

### Proteomic analyses

For protein extraction, 1.5 ml of 1% SDS extraction buffer (1% SDS, 0.1 M Tris/HCl pH 7.5, 10 mM EDTA) was added to unfolded 25 mm filter samples. Each sample was incubated at room temperature (RT) for 15 min, heated at 95 °C for 10 min, and shaken at RT, 350 r.p.m. for 1 h. The protein extract was decanted and placed in a new tube and centrifuged at 14,100*g* (14,500 r.p.m.) for 20 min at RT. The supernatants were removed and concentrated by membrane centrifugation to ∼300 μl in 6 ml, 5 K molecular weight cutoff Vivaspin units (Sartorius Stedim, Goettingen, Germany). Each sample was precipitated with cold 50% methanol (MeOH) 50% acetone 0.5 mM HCl for 3 days at –20 °C, centrifuged at 14,100*g* for 30 m at 4 °C, decanted and dried by vacuum concentration (Thermo Savant Speedvac) for 10 min or until dry. Pellets were resuspended in 1% SDS extraction buffer and left at RT for 1 h to completely dissolve. Total protein was quantified (Bio-Rad DC protein assay, Hercules, CA) with BSA as a standard.

For protein digestion, extracted proteins were purified from SDS detergent, reduced, alkylated and trypsin digested while embedded within a polyacrylamide tube gel^38^. A gel premix was made by combining 1 M Tris-HCl (pH 7.5) and 40% Bis-acrylimide L 29:1 (Acros Organics) at a ratio of 1:3. The premix (103 μl) was combined with an extracted protein sample (35–200 μg), Tris EDTA, 7 μl 1% ammonium persulfate and 3 μl of tetramethylethylenediamine (Acros Organics) to a final volume of 200 μl. After 1 h of polymerization at room temperature 200 μl of gel fix solution (50% ETOH, 10% acetic acid in liquid chromatography/mass spectrometry (LC/MS) grade water) was added to the top of the gel and incubated at RT for 20 min. Liquid was then removed and the tube gel was transferred into a new 1.5 ml microtube containing 1.2 ml of gel fix solution then incubated at RT, 350 r.p.m. in a Thermomixer R (Eppendorf) for 1 h. gel fix solution was then removed and replaced with 1.2 ml destain solution (50% MeOH, 10% acetic acid in H_2_O) and incubated at 350 r.p.m., RT for 2 h. Liquid was then removed, gel cut up into 1 mm cubes and then added back to tubes containing 1 ml of 50:50 acetonitrile:25 mM ammonium bicarbonate (ambic) incubated for 1 h, 350 r.p.m. at RT. Liquid was removed and replaced with fresh 50:50 acetonitrile:ambic and incubated at 16 °C 350 r.p.m. overnight. The above step was repeated for 1 h the following morning. Gel pieces were then dehydrated twice in 800 μl of acetonitrile for 10 min at RT and dried for 10 min in a ThermoSavant DNA110 speedvac after removing solvent. 600 μl of 10 mM dithiothreitol in 25 mM ambic was added to reduce proteins incubating at 56 °C, 350 r.p.m. for 1 h. Unabsorbed dithiothreitol solution was then removed with volume measured. Gel pieces were washed with 25 mM ambic and 600 μl of 55 mM iodacetamide was added to alkylate proteins at RT, 350 r.p.m. for 1 h. Gel cubes were then washed with 1 ml ambic for 20 min, 350 r.p.m. at RT. Acetonitrile dehydrations and speedvac drying were repeated as above. Trypsin (Promega #V5280) was added in appropriate volume of 25 mM ambic to rehydrate and submerse gel pieces at a concentration of 1:20 μg trypsin:protein. Proteins were digested overnight at 350 r.p.m. 37 °C. Unabsorbed solution was removed and transferred to a new tube. 50 μl of peptide extraction buffer (50% acetonitrile, 5% formic acid in water) was added to gels, incubated for 20 min at RT then centrifuged at 14,100*g* for 2 min. Supernatant was collected and combined with unabsorbed solution. The above peptide extraction step was repeated combining all supernatants. Combined protein extracts were centrifuged at 14,100*g* for 20 min, supernatants transferred into a new tube and dehydrated down to ∼10–20 μl in the speedvac. Concentrated peptides were then diluted in 2% acetonitrile 0.1% formic acid in water for storage until analysis. All water used in the tube gel digestion protocol was LC/MS grade, and all plastic microtubes were ethanol rinsed and dried before use.

For MS global proteome analyses, chromatography was performed using a Michrom Advance nanoflow LC and autosampler (Michrom Bioresources) and a 100 μm inner diameter 15 cm long capillary column with a pulled tip (packed in-lab with MAGIC C18AQ 200 Å pore size 3 μm particle size from Michrom Bioresources). Samples were first loaded on a 200 μm I.D. 1 cm long trap (Thermo Scientific Acclaim PepMap100 nano-trap column, 5 μm particle size) and washed with 50 μl of 2% acetonitrile and 0.1% formic acid in water. The trap was then switched in-line with the 15 cm column and eluted with a non-linear gradient of 5–35% solvent B (0.1% formic acid in acetonitrile) balanced with solvent A (0.1% formic acid in water) at 500 nl min^−1^ flow rate.

Electrospray ionization was performed with a Thermo Flex ion source in positive ion mode at 1,400 V. Eluting peptides were analysed on a Thermo Fusion mass spectrometer with MS1 scans at an Orbitrap resolution of 60 K, 350–1,800 m/z scan range, 2.0e5 automatic gain control target, and a maximum injection time of 35 ms. MS2 scans were analysed on the linear ion trap in topN data dependent mode at a cycle time of 3 s using normal scan rate and range with a maximum injection time of 75 ms and a 0.7 m/z isolation window. Charge states of 2–7 were analysed with a dynamic exclusion of 15 s with a mass tolerance of 10 p.p.m. Monoisotopic precursor selection was used, and a user-defined lock mass of 445.12003 *m*/*z*.

Protein identifications were made using the SEQUEST peptide mapping algorithm within Proteome Discoverer and the Peptide Prophet algorithm within Scaffold 3.0 (Proteome Software, Portland, OR, USA) using 99% protein and 95% peptide confidence levels, allowing one minimum peptide per protein, resulting in a 1.1% false discovery rate for proteins and a 0.01% false discovery rate for peptides resulting in 1,499 identified proteins.

Changes in relative abundance of proteins between samples were determined using label-free spectral count enumeration within Scaffold. Spectral counts compare a specific protein's abundance between treatments, rather than against other proteins, because the sensitivity of spectral counts can vary between proteins depending on the number of tryptic peptides within the sequence and their chemical characteristics. These problems do not affect comparisons of a protein with itself between treatments. Spectral counts were normalized within each experimental treatment to the total number of spectra collected to correct for small variations in the number of spectra between samples sets; under stable and consistent MS conditions this results in a very small difference^39^,^40^,^41^. Based on these spectral count enumeration results, the Power Law Global Error Model was used to detect differentially expressed proteins^42^.

### DNA methylation analyses

Cultures were rapidly and gently filtered during the middle of the photoperiod onto 5 μm polycarbonate filters (Whatman), washed off of the filters with 2 ml of 50 mM (pH=7) Tris-HCl into PowerPlant Bead tubes from the PowerPlant Pro DNA Isolation Kit (#13200-100), and put on ice. The tubes were vortexed for 2 min (Vortex Genie 2, Scientific Industries, setting 10), and technical replicates were made for each biological replicate. Protein concentrations were quantified using the Pierce BCA Protein Assay Kit (#23225), and the (Epigentek) Epiquick DNMT Activity/Inhibition Assay Ultra Kit (Fluorometric) (#P-3010) was used to determine DNA methylation activity according the manufacturer's instructions.

## Additional information

**How to cite this article:** Hutchins, D.A. *et al*. Irreversibly increased nitrogen fixation in *Trichodesmium* experimentally adapted to elevated CO_2_. *Nat. Commun.* 6:8155 doi: 10.1038/ncomms9155 (2015).
